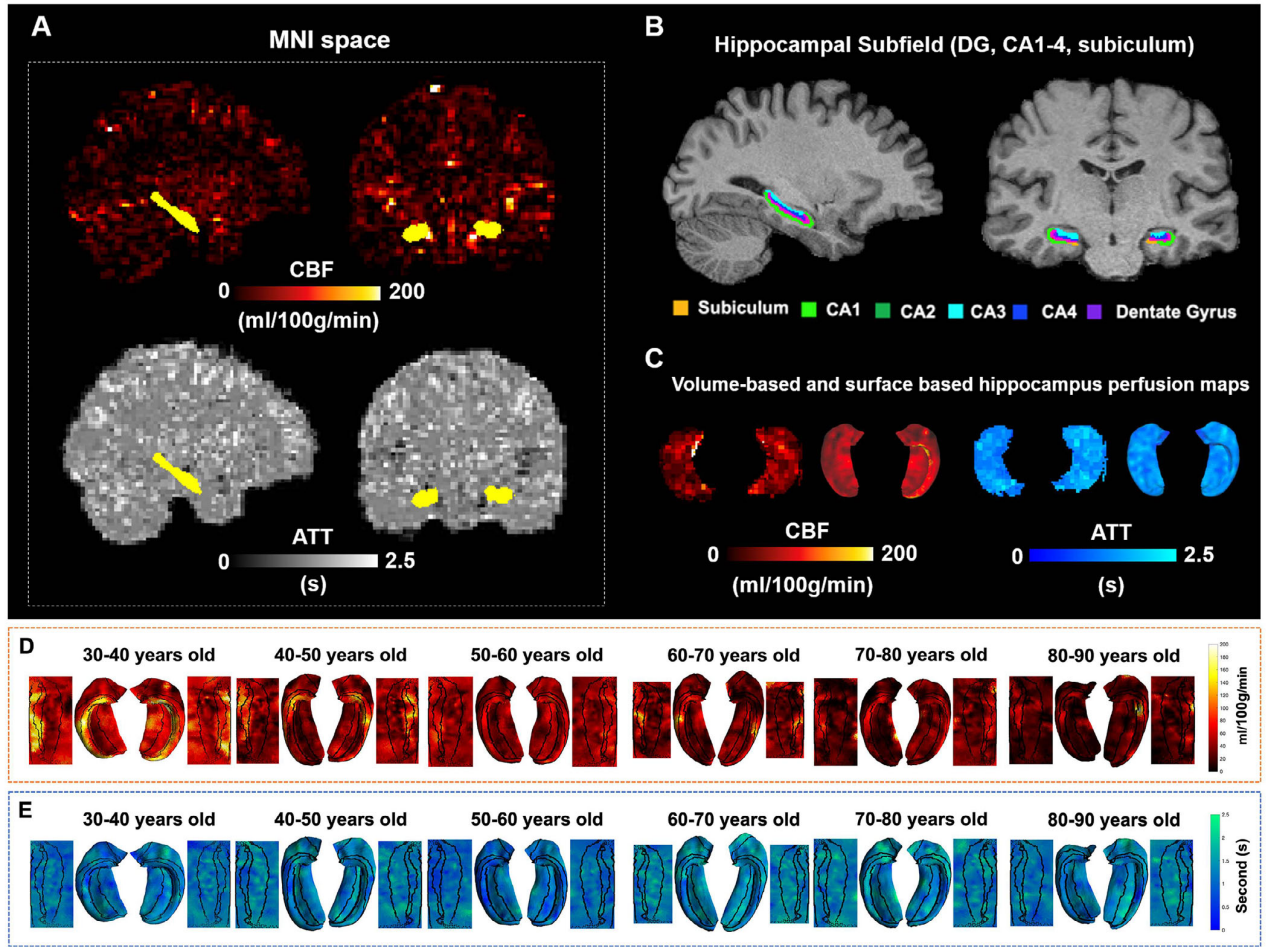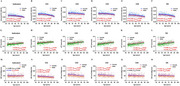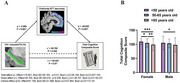# Sex‐specific Trajectories of Structure and Hemodynamics Alterations in Hippocampal Subfields and Connected Cortex During Aging

**DOI:** 10.1002/alz70856_103057

**Published:** 2025-12-25

**Authors:** Jiaqi Wen, Chenyang Li, Zhe Sun, Chao Wang, Jiangyang Zhang, Xiaojun Guan, Xiaojun Xu, Thomas Wisniewski, Yulin Ge

**Affiliations:** ^1^ New York University Grossman School of Medicine, New York, NY, USA; ^2^ The Second Affiliated Hospital, Zhejiang University School of Medicine, Hangzhou, Zhejiang, China; ^3^ NYU Grossman School of Medicine, New York, NY, USA

## Abstract

**Background:**

As a primary structure affected by aging, the hippocampus and its subfields undergo tissue loss and diminished perfusion. However, the relationship between hippocampus structure and vascular function remains unclear. The hippocampus connects with the prefrontal and entorhinal cortex, playing an essential role in cognitive function. Additionally, hormonal changes in older women may heighten their vulnerability to aging, emphasizing the need to investigate sex‐specific patterns in the aging of these interconnected regions. This study aimed to explore the relationship between structural and vascular functional alterations in the hippocampus subfields and connected prefrontal and entorhinal cortex, as well as their influence on cognition during healthy aging.

**Method:**

T1‐MPRAGE and multiple post‐labeling delay pCASL data of 650 subjects from HCP‐Aging dataset was processed. The prefrontal and entorhinal cortex were segmented using the FreeSurfer pipeline. The segmentation of hippocampus subfields, including subiculum, CA1‐4, and dentate gyrus was performed using Hippfold toolbox. The pCASL data was preprocessed using hcpasl minimal processing pipeline. cerebral blood flow (CBF) and aterial transit time (ATT) in hippocampal subfields was unfolded and projected into the hippounfolded space to acquire subfield‐specific values (Figure 1). Total cognitive composite scores were used to reflect overall cognition.

**Result:**

All hippocampal subfields demonstrated age‐related atrophy, with ATT showing greater vulnerability to aging and stronger correlations with atrophy than CBF (Figure 2). Among all hippocampal subfields, the CA1 region displayed the lowest perfusion levels and the strongest association with atrophy. Perfusion in the hippocampal subfields of females appeared more susceptible to aging and atrophy than males. In males, CBF of the subiculum might compensate for the atrophy of this subfield and decreased CBF in other hippocampal subfields. The prefrontal cortex exhibited more pronounced perfusion decline than the entorhinal cortex, and CA1 atrophy impacts cognition through delayed ATT in the prefrontal cortex in females (Figure 3).

**Conclusion:**

Our study revealed the relationship between structure and vascular function in the hippocampus and connected prefrontal and entorhinal cortex, uncovering sex‐ and subfield‐specific trajectories with age. A deeper understanding of these relationships could provide insight into the mechanisms underlying age‐related neurodegenerative diseases such as Alzheimer's.